# Generalized Taylor dispersion of chiral microswimmers

**DOI:** 10.1098/rsta.2024.0262

**Published:** 2025-09-11

**Authors:** Keito Ogawa, Kenta Ishimoto

**Affiliations:** ^1^ Research Institute for Mathematical Sciences, Kyoto University, Kyoto, Japan; ^2^ Department of Mathematics, Kyoto University, Kyoto, Japan

**Keywords:** microswimmers, transport phenomena, Taylor dispersion

## Abstract

Transport phenomena of microswimmers in fluid flows play a crucial role in various biological processes, including bioconvection and cell sorting. In this article, we investigate the dispersion behaviour of chiral microswimmers in a simple shear flow using the generalized Taylor dispersion theory, inspired by biased locomotion of bacterial rheotaxis swimmers. We thus focused on the influence of shear-induced torque effects due to particle chirality, employing an extended Jeffery equation for individual deterministic dynamics. We then numerically calculated macroscopic parameters, including the average swimming velocity and the effective diffusion tensor using spherical harmonic expansion, and evaluated the obtained results based on the fixed points and the stability of the orientational dynamical systems. Our results reveal that chiral effects induce biased locomotion, and we observed qualitative transitions in the orientational distribution with increasing Péclet number, consistent with previous experimental findings. The diffusion tensor analysis highlighted a significant reduction in the diffusion coefficient perpendicular to the shear plane due to chirality. This suggests potential applications in flow-mediated cell separation, and we numerically demonstrated such chirality-induced fluid transport.

This article is part of the theme issue ‘Biological fluid dynamics: emerging directions’.

## 1. Introduction

Transport of particles in a fluid flow has been an important research subject in biological fluid dynamics for understanding and controlling mixing, diffusion and separation processes of colloidal particles and suspensions, including cancer and red blood cells [1,2]. The past two decades have seen research interest in the transport of self-propelled particles and suspensions of such particles [3–5], being motivated by high-throughput image data of swimming bacteria and eukaryotic plankton, as well as precise manipulation of these cells by microfluidic devices.

In the fluid transport of these microswimmers, their orientational dynamics becomes more significant in addition to the dynamics of particle position, because it determines the swimming direction and directly affects particle transport. Many biological swimmers in a fluid flow then exhibit various trajectory patterns as a result of their orientational dynamics and active motion. Indeed, in the vicinity of a wall boundary, some swimming cells have been known to swim upstream against a flow, which is often referred to as rheotaxis [6–8].

In a bulk shear flow, in contrast, bacterial cells exhibit migration perpendicular to the shear plane, which is called bacterial bulk rheotaxis [9–12]. A spheroidal particle in an inertia-less regime periodically rotates in a simple shear flow, and its orientational vector forms a closed loop known as a Jeffery orbit [13]. This periodic rotation is described by an exact solution to the Stokes equation for a low-Reynolds-number flow. Recent studies found that the key physics behind bacterial bulk rheotaxis is the chirality of the particle, which is not considered in the Jeffery orbits. Indeed, swimming bacterial cells possess helical appendages called flagella and such a chiral object experiences a fluid torque, turning the cells away from the shear plane, that is, to be aligned parallel to the background vorticity vector. This chirality-induced torque was recently theoretically formulated through hydrodynamic shape theory, which is based on the invariant properties of resistance tensors under a particular change of coordinates [14,15]. Ishimoto [16,17] considered an arbitrary axisymmetric hydrodynamic shape and extended the Jeffery equation into chiral particles, which include a simple helix and bacterial cells.

**Figure 1.**
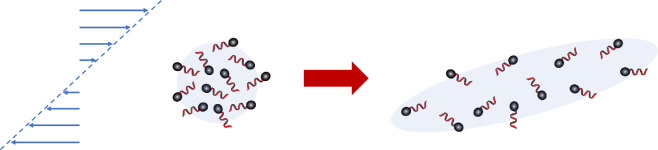
Schematic of Taylor dispersion of chiral microswimmers in a shear flow.

As illustrated in figure 1, these chiral microswimmers are transported in a fluid flow and, at the same time, they disperse due to thermal and active orientational diffusion. To analyse these stochastic processes, we consider a probability distribution function in physical and orientation phase space, which is, in general, six-dimensional, posing a numerical challenge [18]. Here, focusing on a macroscopic diffusion process of chiral microswimmers, we used the generalized Taylor dispersion (GTD) theory [19,20] that bypasses large-dimensional computations to obtain lower order statistical moments, including the diffusion constant.

The GTD theory has been applied to the analysis of population-level dispersion behaviour of swimming microorganisms in shear flows, particularly in the context of bioconvection [3]. Hill & Bees [21] considered a spherical microswimmer that exhibits a gyrotactic response to swim upwards in a simple shear flow under gravity. Bearon [22] analysed the macroscopic diffusion of spherical microswimmers with chemotactic responses. Manela & Frankel [23] comprehensively analysed non-spherical microswimmers with gyrotactic response to estimate the macroscopic diffusion tensor, pointing out numerical difficulty at high Péclet numbers, where the shear strength dominates the individual swimmer diffusion. The GDT theory has also been used for chemotactic microswimmers [22,24,25], emphasizing the wide applicability of the approach.

In this study, inspired by bacterial bulk rheotaxis, we examined the population-level dispersion phenomena for chiral microswimmers in a shear flow by using the GTD theory to quantitatively estimate the macroscopic transport velocity of the distribution and its diffusion coefficients. Using the extended Jeffery equation for particle dynamics in a simple shear flow, we developed a general theoretical framework and numerical demonstrations that quantify the dispersive transport of chiral swimmers.

## 2. Problem set-up

### (a) Chiral active particles in a simple shear flow

**Figure 2.**
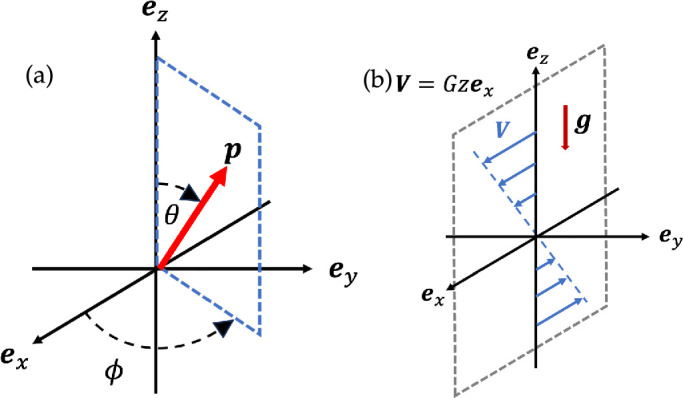
Schematic of coordinates and shear flows. (a) Polar coordinates for the particle orientation vector p. (b) External shear flow set as $V=Gze_{x}$ with its shear strength G. Gravity g is taken as downwards (negative $e_{z}$ axis).

We consider a microswimmer that is moving along its orientation vector p with speed $V_{s}$ in a quiescent flow. As in figure 2a, we introduce the laboratory frame $\left\{e_{x},e_{y},e_{z}\right\}$ and polar coordinates (θ,ϕ) to describe the swimmer orientation vector p. We assume that the particle is located in a linear background flow V(x) with $x=(x,y,z{)}^{\text{T}}$. The background vorticity vector $\omega_{i}=\epsilon_{ijk}(\partial V_{k}/\partial x_{j})$ (i,j,k∈{x,y,z}) and the rate-of-strain $E_{ij}=(\partial V_{i}/\partial x_{j}+\partial V_{j}/\partial x_{i})/2$ are constants over the space, where $\epsilon_{ijk}$ is the Levi–Civita symbol. The Einstein summation convention is used throughout this article.

We assume that the swimmer is neutrally buoyant but that the centre of geometry does not always coincide with the centre of gravity, as usually assumed for gyrotactic swimming microorganisms. As in figure 2b, we take the gravitational acceleration towards $-e_{z}$ axis, and the background flow as a simple linear shear flow via $V=Gze_{x}$. This flow configuration has been studied in the context of swimming algae in the upper ocean and is referred to as a vertical shear flow [21].

The angular dynamics of a microscopic spheroidal object in a linear background flow follows the well-known Jeffery equation [13]. Chiral objects, such as a helix, experience shear-induced hydrodynamic forces and torques, and recently, the Jeffery equation was extended to such chiral objects, given by [16]


(2.1) $$\begin{array}{r} \dot{p}=\frac{1}{2\tau_{g}}\left[e_{z}-(e_{z}\cdot p)p\right]+\frac{1}{2}\omega \times p+b(I-pp)\cdot E\cdot p+c\left[(I-pp)\cdot E\cdot p\right]\times p, \end{array}$$

where the dot symbol on the left-hand side denotes the time derivative and I is the identity tensor. The first term on the right-hand side of equation (2.1) represents the alignment towards the $e_{z}$ axis by the gravitational torque and $\tau_{g}$ is the time scale for this alignment. The second term is the rotation due to local background vorticity. The third and fourth terms include the shape effects.

The parameter b represents the effective aspect ratio and is known as the Bretherton parameter [26]. This value typically ranges from −1 to 1, with b=0 for a sphere, b→1 at the slender limit and b→−1 for the disc limit. The parameter c represents the strength of the chirality-induced rotation and thus vanishes for an achiral particle, such as a revolving body. In a simple shear flow, a chiral particle experiences a drift force perpendicular to the shear plane. Hence, if the particle chirality is homogeneous along its body such as a simple helix, no chirality-induced torque is generated [27,28]. When the particle chirality does not possess the fore-aft asymmetry, such as a bacterial cell, the chirality-induced transverse force then generates torque that turns the object towards perpendicular to the shear plane. The parameter c is therefore interpreted as chirality moment rather than chirality itself [16]. According to recent observations and theoretical calculations, the parameter c ranges |c|∼0.01−0.1 for swimming bacteria with helical flagella and a cell body [7,10,16]. The sign of c represents the sign of the chirality, and c>0 corresponds to a bacterium with a left-handed helix such as *E. coli*.

Particle chirality also induces shear-induced drift velocity $U_{s}(p)$, and the total velocity is the sum of the background flow as $U=Gze_{x}+U_{\text{s}}$. Its general form is explicitly given by [16,17], with extra terms containing shape parameters as in equation (2.1). For a typical bacterial swimmer, however, these additional drift terms are significantly smaller compared with the swimming velocity [29]. Hence, we neglect these additional terms as in the previous study [10], and we simply set $U_{s}=V_{s}p$ with $V_{s}$ being the swimming velocity.

We introduce the probability distribution function for a swimmer whose position and orientation are, respectively, $R^{\prime }$ and $p^{\prime }$ at time t=0 and R and p at time t by $P(R,p,t|R^{\prime },p^{\prime })$. The Fokker–Planck equations for this distribution therefore satisfy $(\partial P/\partial t)+\nabla_{R}\cdot J+\nabla_{p}\cdot j=0,$ with $\nabla_{R}$ and $\nabla_{p}$ denoting the gradients in space and orientation, defined as $\nabla_{R}=e_{x}\frac{\partial }{\partial x}+e_{y}\frac{\partial }{\partial y}+e_{z}\frac{\partial }{\partial z}$ and $\nabla_{p}=e_{\theta }\frac{\partial }{\partial \theta }+e_{\phi }\csc\;\theta \frac{\partial }{\partial \phi }$, respectively. The probabilistic current in space and orientation is obtained as $J=U(R,p)P\text{and}j=\dot{p}(p)P-d_{r}\nabla_{p}P,$ where $d_{r}$ is the rotational diffusion constant and we neglect the translational diffusion for brevity, as this effect is less important in many swimming microorganisms compared with the rotational diffusion [21].

### (b) Generalized Taylor dispersion theory

By writing the mth moment of the distribution P as an m-rank tensor,


(2.2) $$\begin{array}{lrr} & M_{m}=\int_{{\mathbb{R}}^{3}}\int_{S^{2}}(R-R^{\prime }{)}^{m}Pd^{2}pd^{3}R, & \end{array}$$

we may define the average velocity and the effective diffusion tensor via the first and second moments as


(2.3) $$\begin{array}{lcr} & \bar{U}=\lim_{t\to \infty }\;\frac{\delta M_{1}}{\delta t}\;\text{and}\;\;\bar{D}=\lim_{t\to \infty }\;\frac{1}{2}\frac{\delta }{\delta t}(M_{2}-M_{1}M_{1}), & \end{array}$$

respectively. Here, the derivative represents the Oldroyd derivative in the co-deforming frame with the fluids, given by $\delta A/\delta t=DA/Dt-G\cdot A-A\cdot G^{\text{T}}$ for an arbitrary tensor A, where D/Dt represents the Lagrangian (material) derivative and G is the velocity gradient tensor whose components are given by $G_{ij}=\partial V_{j}/\partial x_{i}$. We introduce the spatial average of the probability distribution $P_{0}$ and its long-time asymptotic form as


(2.4) $$\begin{array}{lcr} & P_{0}=\int_{{\mathbb{R}}^{3}}P(R,p,t|R^{\prime },p^{\prime })d^{3}R\;\;\text{and}\;\;P_{0}^{\infty }(p)=\lim_{t\to \infty }\;\int_{{\mathbb{R}}^{3}}P(R,p,t|R^{\prime },p^{\prime })d^{3}R. & \end{array}$$

According to the GTD theory [19,30], the average velocity and the effective diffusion constant are provided as


(2.5) $$\begin{array}{lcr} & \bar{U}=\int_{S^{2}}P_{0}^{\infty }(p)U_{s}(p)d^{2}p\;\text{and}\;\;\bar{D}=\int_{S^{2}}[V_{s}P_{0}^{\infty }Bp+P_{0}^{\infty }BB\cdot G{]}^{\text{sym}}d^{2}p, & \end{array}$$

where ‘sym’ indicates its symmetric part, and the vector field B(p) is introduced as the long-time limit of the spatial deviation of a particle with its orientation p obtained from its mean, given by


(2.6) $$\begin{array}{lcr} & B(p)=\lim_{t\to \infty }\left(\frac{{\bar{P}}_{1}}{P_{0}}-M_{1}\right)\;\text{with}\;\;{\bar{P}}_{1}(p,t|p^{\prime })=\int_{{\mathbb{R}}^{3}}(R-R^{\prime })Pd^{3}R. & \end{array}$$

To non-dimensionalize the system, we use the shear strength as the unit of time, that is, $T=G^{-1}$. We employ $L=V_{s}/d_{r}$ as the unit of length, which naturally introduces the non-dimensional parameter of the system, known as the Péclet number: $\text{Pe}=G/d_{r}$. With $b=P_{0}^{\infty }Bd_{r}/V_{s},\hat{G}=G/G$ and $\hat{\dot{p}}=\dot{p}/G$, the equations for $P_{0}^{\infty }$ and b are provided by the GTD theory as


(2.7) $$\begin{array}{lcr} & \nabla_{p}\cdot (\text{Pe}\dot{p}P_{0}^{\infty }-\nabla_{p}P_{0}^{\infty })=0\;\text{with}\;\;\int_{S^{2}}P_{0}^{\infty }d^{2}p=1, & \end{array}$$

and


(2.8) $$\begin{array}{lrr} & \nabla_{p}\cdot [\text{Pe}\hat{\dot{p}}b-\nabla_{p}b]-\text{Pe}b\cdot \hat{G}=P_{0}^{\infty }(p-\bar{p})\text{with}\int_{S^{2}}bd^{2}p=0. & \end{array}$$

To obtain the values of the coefficients, we set up the equations to solve equations (2.7) and (2.8). Substituting the expression for the background flow field $V=(Gz,0,0{)}^{\text{T}}$ leads to the angular dynamics of the chiral particle, with a non-dimensionalized gyrotactic time scale $g=1/\tau_{g}G$, given by



(2.9)θ˙=−g2sin⁡θ+12(1+bcos⁡2θ)cos⁡ϕ−c2cos⁡θsin⁡ϕ,(2.10)ϕ˙sin⁡θ=−12(1+b)cos⁡θsin⁡ϕ−c2cos⁡2θcos⁡ϕ.

We further expand this set of equations with the spherical harmonics and rewrite the system as a set of linear relations between the coefficients of the spherical harmonics. Some details are shown in the electronic supplementary material. Hill & Bees [21] computed the GTD for a spherical microswimmer with a truncation $n_{\text{max}}=4$. For non-spherical swimmers and chiral swimmers, we need higher order terms in the spherical harmonics to guarantee their numerical accuracy. In this study, we analysed this by changing the series truncation from $n_{\text{max}}=10$ to $n_{\text{max}}=30$, depending on the parameter sets.

## 3. Orientational dynamics and its distribution

Before we proceed to the numerical results of the population-level distribution, it is beneficial to examine the deterministic angular dynamics described by equations (2.9) and (2.10). These equations possess a symmetry under the changes of c↦−c and ϕ↦−ϕ, and we focus on the c>0 case. The fixed points of the dynamics, where $\dot{\theta }=\dot{\phi }=0$, are readily obtained as


(3.1) $$\begin{array}{lcr} & \tan\;\phi =-\frac{c\;\cos\;2\theta }{(1+b)\;\cos\;\theta }\;\text{and}\;\;f_{\pm }(\theta )=0, & \end{array}$$

where $f_{\pm }(\theta )$ are given by


(3.2) $$\begin{array}{lrr} & f_{\pm }(\theta )=-g\;\sin\;\theta {\left[(1+b{)}^{2}\cos^{2}\;\theta +c^{2}\cos^{2}\;2\theta \right]}^{1/2}\pm \cos\;\theta \left[c^{2}\cos\;2\theta +(1+b)(1+b\;\cos\;2\theta )\right]. & \end{array}$$

If the gyrotactic effects are negligible (g=0), these fixed points are represented simply. When $b^{2}+c^{2}\leq 1$, there are only two fixed points, given by (ϕ,θ)=(±π/2,π/2). When $b^{2}+c^{2}>1$, however, there are six fixed points:


(3.3) $$\begin{array}{lrr} & (\phi ,\theta )=\left(\pm \frac{\pi }{2},\frac{\pi }{2}\right),\;(\phi_{1},\theta_{1}),\;(\phi_{1}-\pi ,\theta_{1}),\;(-\phi_{1},\pi -\theta_{1}),\;(\pi -\phi_{1},\pi -\theta_{1}), & \end{array}$$

where $\phi_{1}$ and $\theta_{1}$ are given by


(3.4) $$\begin{array}{lcr} & \phi_{1}=\arctan\;\sqrt{\frac{2c^{2}}{\left(b^{2}+b+c^{2})(b^{2}+c^{2}-1\right)}}\;\text{and}\;\;\theta_{1}=\arccos\;\sqrt{\frac{b^{2}+c^{2}-1}{2(b^{2}+b+c^{2})}}, & \end{array}$$

respectively.

**Figure 3.**
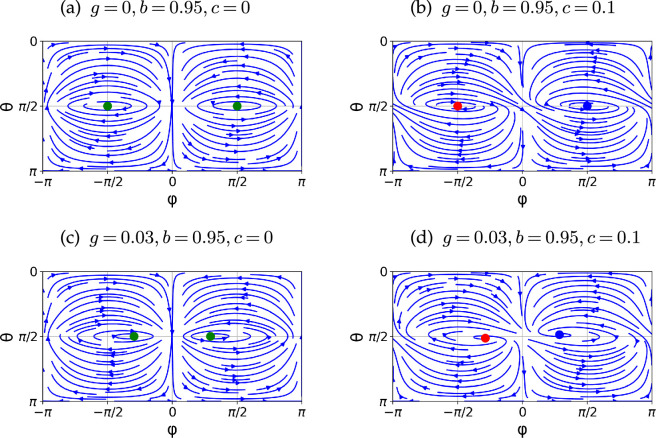
Phase portraits of the generalized Jeffery equation for a chiral particle under a gyrotactic torque g with different shape parameters b and c. Streamlines in the ϕ−θ plane are shown and the fixed points are marked with different colours and symbols (blue circle: attractive fixed point; red circle: repelling fixed point; green circle: neutrally stable and green diamond: saddle fixed point). (a) Periodic motion known as the Jeffery orbit. (b) Attractive and repelling fixed points emerging due to the chirality ($b^{2}+c^{2}<1)$. (c) The gyrotactic torque only modulates the periodic orbits at a small g with g<1−b. (d) The gyrotactic torque also modulates the position of attracting and repelling fixed points for a chiral particle.

In figure 3, we present streamlines and the stability of these fixed points with different gyrotactic and shape parameters. Throughout the article, to focus on biologically relevant situations, we consider g=0.03 for typical gyrotactic time scales, as seen in Chlamydomonas [3,31], and for shape parameters, we consider b=0.95 and c=0.1 for typical chiral microswimmers, following recent experiments with E.coli bacteria by Jing *et al.* [10].

For a non-chiral particle (c=0), the angular dynamics has two fixed points. We found that, when g>1−b, one of the fixed points located in 0<θ<π/2 is linearly stable and the other fixed point is linearly unstable. When g≤1−b, these two fixed points become neutrally stable. As shown in figure 3a,b, a chiral particle with c>0 and g=0 possesses one attractive and one repelling fixed point in the phase portrait, while the non-chiral particle (g=c=0) follows the periodic motions of the Jeffery orbit. A linear stability analysis of these fixed points leads to zero real parts of the eigenvalues, and attraction and repelling dynamics are known to be slower than exponential in time and scale as $t^{-1/2}$ [16]. When c increases from c=0.1, the attraction and repulsion of the fixed points in figure 3b become stronger as far as $b^{2}+c^{2}<1$ is satisfied. When the sign of c is reversed, the stability of the fixed points is also reversed. In many biological swimmers, the gyrotactic torque is sufficiently weak to satisfy g<1−b and only modulates the angular dynamics to attract the swimmer towards ϕ=0, which corresponds to the flow direction (figure 3c,d).

**Figure 4.**
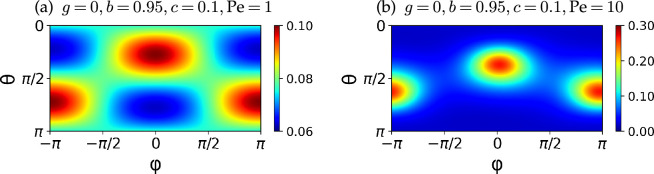
Probability distributions of the orientation $P_{0}^{\infty }(\theta ,\phi )$ at (a) Pe=1 and (b) Pe=10 for chiral particles with g=0,b=0.95andc=0.1. At a small Pe, the distribution is almost symmetric to ϕ=0 and the chiral effects are irrelevant.

We now present our numerical results on the probability distribution of the particle orientation $P_{0}^{\infty }(\theta ,\phi )$. When Pe is small, the distribution is almost uniform in θ−ϕ space. By expanding the series, $P_{0}^{\infty }=(1/4\pi )+\text{Pe}P_{1}^{\infty }+{\text{Pe}}^{2}P_{2}^{\infty }+\cdots \;,$ and plugging it into equation (2.7), the O(Pe) term reads the Poisson equation on a unit sphere as


(3.5) $$\begin{array}{lcr} & \nabla_{p}^{2}P_{1}^{\infty }=\frac{1}{4\pi }\nabla_{p}\cdot \dot{p}=\frac{1}{4\pi }\left(-g\;\cos\;\theta -3b\;\sin\;\theta \;\cos\;\theta \;\cos\;\phi \right), & \end{array}$$

where the source term on the right-hand side is independent of the chirality effects. This chirality independence is also seen in figure 4, where we show the probability distribution $P_{0}^{\infty }$ at small Pe (Pe=1 and Pe=10). As can be seen, the probability distribution is almost symmetric about ϕ=0, even for chiral particles.

**Figure 5.**
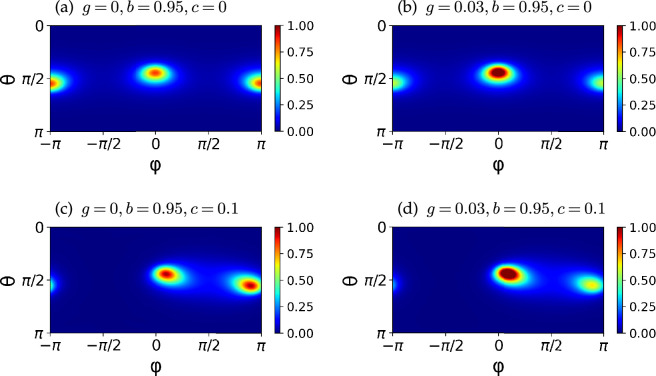
Probability distributions of particle orientation $P_{0}^{\infty }(\theta ,\phi )$ at Pe=100 with different gyrotactic and shape parameters. (a) The particle orientation is symmetrically localized at ϕ=0,π in the absence of gyrotactic and chirality effects. (b) If only the gyrotactic torque is applied, g>0, the orientation becomes biased at ϕ=0. (c) If only the chirality effect is considered, the particle orientation is bimodally distributed around ϕ=π/2. (d) When both the gyrotactic and chirality effects are considered, the distribution becomes localized around one of the peaks near ϕ=0.

In figure 5, we show colour maps of $P_{0}^{\infty }(\theta ,\phi )$ with different gyrotactic and shape parameters at Pe=100. When the particles are non-chiral, the orientation distribution is still symmetric about ϕ=0 at the higher value of Pe, as in the deterministic dynamics in figure 3. When the particle is chiral, however, there is an attractive fixed point in the θ−ϕ phase space, which breaks the symmetry about ϕ=0. Hence, as Pe increases, the distribution of the orientation of a chiral particle accumulates at the attracting fixed point in the θ−ϕ space (figure 3). Nonetheless, because the attraction is weaker than an exponential function in time, even with a large Pe, the orientation probability is distributed around the attracting points, as seen in figure 5c,d.

In particular, when g=0 a chiral particle exhibits a bimodal distribution around ϕ=π/2 (figure 5c). This result is in agreement with experiments and stochastic simulations by Jing *et al.* [10], where the rotational diffusion was set as $d_{r}=0.057$ s${,}^{-1}$ for E.coli bacteria and hence $\text{Pe}=G/d_{r}\approx 17.54\times G$. Jing *et al.* present the simulation results with G=1,10,100,1000 s${,}^{-1}$ and a bimodal distribution at an intermediate shear strength with G=10 s${,}^{-1}$. We also observed the accumulation of the bimodal distribution into a single-peak distribution as c increased, reflecting the stronger attraction towards the stable fixed point.

The gyrotactic torque (g>0) attracts the orientation around ϕ=0 as predicted from the analysis of the deterministic dynamical systems. Figure 5d shows that the distribution becomes localized at the left peak near ϕ=0 from the bimodal distribution of figure 5c.

**Figure 6.**
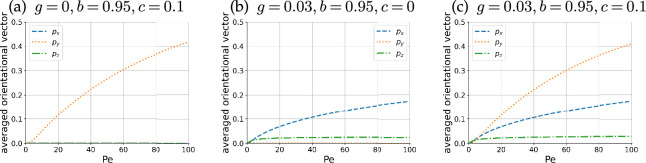
Components of average orientation $p_{x},p_{y},p_{z}$ with different Pe values with 0≤Pe≤100. (a) Chiral particle with c>0 moves towards the background vorticity vector ($p_{y}>0$), while the symmetry in the x and z axes results in zero mean components, irrespective of the value of Pe. (b) Gyrotactic torque breaks the symmetry in the x and z axes but holds the symmetry in the y-axis, yielding non-zero mean values only for $p_{x}$ and $p_{z}$. (c) Effects of gyrotactic torque and chiral torque exhibiting symmetry breaking to generate non-zero mean values in each component almost independently.

We then computed the average orientation $\bar{p}$ with different Pe values to create the plots in figure 6; the orientation is proportional to the average swimming velocity obtained by $\bar{U}=(4\pi /3)V_{s}\bar{p}$. The plots for a chiral microswimmer (c>0) without gyrotactic torque show a non-zero mean in the y direction, indicating a swimming velocity transverse to the shear plane, which is compatible with the bacterial bulk rheotaxis (figure 6a). When Pe increases, as the orientational distribution is attracted towards the stable fixed point, the transverse components increase on average. The change in the sign of c results in an additional minus sign for $p_{y}$, which indicates the opposite transverse direction to the shear plane. The gyrotactic torques break the symmetry in the orientational distribution in the x and z directions, yielding non-zero mean values in $p_{x}$ and $p_{z}$ (figure 6b). The chirality and gyrotactic effects are almost independent in this parameter set, as shown in figure 6c.

## 4. Dispersion behaviour

**Figure 7.**
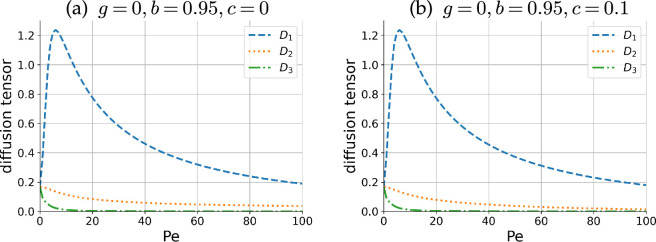
Eigenvalues of the diffusion tensor for (a) non-chiral and (b) chiral particles with different Pe values in 0≤Pe≤100. The diffusion in the $D_{2}$-axis is strongly suppressed due to the chirality effects in the high Pe region.

We then computed equation (2.8) to obtain b and then the diffusion tensor from equation (2.5). In figure 7, we show numerical plots of the eigenvalues of the diffusion tensors for non-chiral and chiral particles with different Pe values. With the correction term, the diffusion tensor is kept positive-definite.

In both panels of the figure, the eigenvalue $D_{1}$ is the largest and $D_{3}$ is the smallest. The corresponding eigenvectors are almost aligned along the x and z axes, respectively. This result is in agreement with the classical Taylor dispersion in that the distribution is strongly distorted towards the flow direction, and we found that the chirality does not alter the dispersion dynamics in the shear plane. In contrast, the eigenvalue $D_{2}$, whose eigenvector is directed to the y-axis, is strongly suppressed due to the chirality effect. With the parameter values in the figure, the suppression is more enhanced as Pe increases. For example, at Pe=100, the value of $D_{2}$ is decreased to 38% for the parameter set used in figure 7. This may be physically interpreted as the focus of the distribution function in the θ−ϕ plane by the attracting fixed point for a large Pe value. The effect of the size of c is also consistent with the θ−ϕ dynamics. The larger |c| values strengthen the attraction to the stable fixed point and the diffusion constant $D_{2}$ is further suppressed.

The gyrotactic torque, however, does not qualitatively alter the eigenvalues. Indeed, when g=0.03, the plots of the eigenvalues are almost identical, and the difference is not visible (figure not shown), which is distinct from the apparent gyrotactic effects in the average orientation of figure 5. This difference may be understood by the fact that the gyrotactic torque breaks the symmetry of the dynamical system but retains the stability of the fixed points.

**Figure 8.**
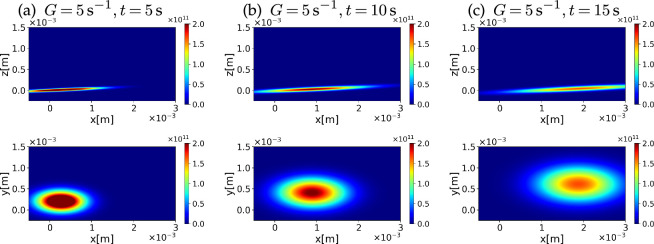
Snapshots of the distribution function for chiral microswimmers in simple shear flow at different times of (a) t=5 s, (b) t=10 s and (c) t=15 s, in the in-flow plane (x−z cross section, top) and the x−y cross section (bottom). The parameter values are g=0.03,b=0.95,c=0.1,Pe=100 and $G=5\;{\text{s}}^{-1}$.

The GTD theory assumes a Gaussian distribution for the population dynamics of microswimmers. With numerically obtained macroscopic model parameters $\bar{p}$ and $\bar{D}$, which are both constants independent of position and time, we may estimate the dispersion behaviour in physical space. We set the initial position of the swimmers at the origin without loss of generality. In figure 8, we show snapshots of the distribution of chiral microswimmers with g=0.03,b=0.95 and c=0.3 at Pe=100 with different times t. The cross sections in the *x–y* and *x–z* planes were chosen to contain the mean of the distribution. To illustrate the physical realization of the population dynamics in a laboratory experiment, we set $V_{s}=10^{-4}\;\text{m/s}$ and $G=5\;{\text{s}}^{-1}$ with t=5,10,15s and visualized the distribution function as a colour map with physical units. In the top panel of figure 8, we display the population of microswimmers in the x−z cross section, on which a simple shear flow is applied. The swimmers are driven down by the shear, and the distribution exhibits a large dispersion along the flow direction. The bottom panel of figure 8 shows the same snapshots but in the x−y cross section. The transverse velocity of the chiral microswimmers moves the population towards the y-axis. The suppression of the diffusion in the y-axis also contributes to focused rheotactic transport of the collection of microswimmers. If a swimmer possesses the opposite chirality (c<0), the swimming direction reverses. Hence, a mixture of chiral swimmers with opposite chirality could be easily sorted by this simple shear flow.

## 5. Conclusion

In this study, we numerically investigated the dispersion phenomena for chiral microswimmers in a vertical shear flow by the GTD theory, focusing on the shear-induced torque effects due to the chirality of the particles. A spherical harmonic expansion allowed us to calculate macroscopic parameters for population-level swimmer transport, including the average swimming velocity and effective diffusion tensor. In particular, in a regime with high Pe values, higher order spherical harmonics were necessary to tackle non-spherical, chiral particles, which individually obey an extended Jeffery equation with particle chirality. With the chiral effects of the swimmer shape, the shear-induced torque aligned the swimmer towards the background vorticity, which led to perpendicular migration to the shear plane, as is well known for bacterial bulk rheotaxis. This biased locomotion is well understood by the individual deterministic dynamical systems as an attracting fixed point in orientational phase space. When the Péclet number Pe was small ($\text{Pe}≲O(10^{1})$), however, the chiral torques were masked by individual rotational diffusion, and the orientational distribution was almost symmetric about the shear plane. As the Péclet number increased ($\text{Pe}=O(10^{2})$), however, the distribution became biased to exhibit bimodal peaks around the attracting fixed point until it accumulated to a single peak at a large Pe value. The qualitative transitions are in good agreement with experimental and particle-based numerical simulations of *E. coli* bacteria [10].

With correction terms from the background shear, we were able to guarantee the positive-definiteness of the diffusion tensor and found that the diffusion coefficient $D_{2}$ in the direction perpendicular to the shear plane was remarkably reduced by the chiral effect, which suggests a flow-mediated cell separation depending on the sign of the particle chirality. Indeed, the GTD theory also enabled us to reconstruct the population-level distribution, and our numerical simulations with typical parameter sets demonstrated that microswimmers can be sorted based on their chirality. These results emphasize the usefulness of the GTD theory for predicting and controlling the population-level dispersion behaviour of swimmers and for designing a tailor-made microdevice for cell sorting. Although we only considered a vertical shear case in this study, our numerical method is applicable to a general gravitational direction, including horizontal shear flow [32] and a shear flow near a vertical plume in bioconvection [33], providing interesting future work.

In this study, we have focused on physically and biologically relevant parameter regimes with $b^{2}+c^{2}<1$. The possible values of |c|, however, have not been well understood because of non-trivial relations between the actual physical shape and hydrodynamic shape parameters c [29,34,35]. A particle with $b^{2}+c^{2}\geq 1$ exhibits further complicated dynamics in the θ−ϕ plane and the transport phenomena of such a particle are qualitatively different. We have also neglected the chirality-induced drift terms, because these are significantly small compared with a particle’s swimming velocity. For a passive particle, however, the chirality-induced drift terms must be included, and indeed, this drift is considered to separate particles with opposite chirality [27,28,36,37]. Nonetheless, the GTD theory is formulated for a linear, non-extensional flow, and application to other complex flows, such as a Poiseuille flow, needs special care. Bees & Croze [24,33] studied its application to a pipe flow and found that the GTD theory was validated when the swimming velocity and shear variance were sufficiently small. Bearon *et al.* [38] examined an extension of the GTD theory in an extensional flow by comparing it with stochastic simulation, although the theory can accurately predict the collective behaviour only in one-dimensional flow. More recently, Fung *et al.* [39,40] proposed using the local swimming velocity and local diffusion tensor, instead of modelling the dispersion by a global macroscopic parameter. The dispersion of chiral microswimmers in such flows is practically important for designing microdevices and understanding large-scale dynamics such as bioconvection, which calls for further study.

## Data Availability

Materials for generating the data are provided in the electronic supplementary material [41].
